# Epitaxial Growth of GaN on Magnetron Sputtered AlN/Hexagonal BN/Sapphire Substrates

**DOI:** 10.3390/ma13225118

**Published:** 2020-11-13

**Authors:** Jinxing Wu, Peixian Li, Shengrui Xu, Xiaowei Zhou, Hongchang Tao, Wenkai Yue, Yanli Wang, Jiangtao Wu, Yachao Zhang, Yue Hao

**Affiliations:** 1 Wide Bandgap Semiconductor Technology Disciplines State Key Laboratory, School of Advanced Materials and Nanotechnology, Xidian University, Xi’an 710071, China; jinxing_wu_xidian@163.com (J.W.); pxli@mail.xidian.edu.cn (P.L.); yuewenkai888@gmail.com (W.Y.); ylwang055065@163.com (Y.W.); 2 Wide Bandgap Semiconductor Technology Disciplines State Key Laboratory, School of Microelectronics, Xidian University, Xi’an 710071, China; hchtao@stu.xidian.edu.cn (H.T.); aweaomeivan@gmail.com (J.W.); ychzhang@xidian.edu.cn (Y.Z.); yhao@xidian.edu.cn (Y.H.)

**Keywords:** h-BN, sapphire, magnetron sputtered AlN, GaN

## Abstract

Magnetron sputtering is adopted to deposit ~25 nm thick AlN on the surface of hexagonal BN(h-BN)/sapphire substrates, followed by epitaxial GaN growth on top of the AlN/h-BN/sapphire substrate using a metal–organic chemical vapor deposition system. Compared to GaN grown on the h-BN/sapphire surface directly, this method results in a continuous and smooth GaN film with a smaller root mean square roughness. Besides, the introduction of the sputtered AlN layer reduces the dislocation density of GaN by 35.7%. We provide a pathway of GaN epitaxy on the h-BN surface, which significantly improves its surface morphology and crystal quality.

## 1. Introduction

Devices manufactured on III-nitride materials are widely being applied in the military, industry, and our daily lives [1,2,3,4]. GaN-based light-emitting diodes (LEDs) are used in lighting, backlighting, and decoration [5,6]. Additionally, GaN-based high electron mobility transistors (HEMTs) are adopted in radio frequency and power devices [7]. To obtain III-nitride materials, substrates including sapphire, silicon, and SiC are mostly utilized. The III-nitride material forms a strong covalent *sp*^3^ bond with the substrate, and single-crystal GaN cannot be grown on a polycrystalline or amorphous substrate [8]. However, once the epitaxial layer can be released from the substrate, it is a promising solution to the limitations of the substrate. Traditional techniques including laser lift-off and chemical methods must break the covalent bond between the III-nitride material and the substrate, thus causing material damage, and the process is also time-consuming [9,10].

The van der Waals epitaxy of III-nitrides on two-dimensional materials has been a hot research topic in recent years [11,12,13,14,15,16,17,18,19]. The two-dimensional material h-BN serves as an ideal layer for mechanically releasing devices from the substrate [20]. Since there are no dangling bonds on the surface of the two-dimensional material h-BN or graphene, III-nitrides will form clusters on the surface [21]. It is rather hard to grow III-nitride materials directly on the surface of two-dimensional materials [22]. To overcome this problem, some approaches have been proposed. Wu et al. treated h-BN with O plasma to produce dangling bonds on its surface [23]. The dangling bonds on the h-BN surface facilitate the nucleation of the AlN epitaxial layer. Based on this method, UV-LED devices with an emission wavelength of ~281 nm were successfully achieved [24]. Graphene treated with N plasma introduces dangling bonds on its surface. Dangling bonds are more conducive to the nucleation of AlN. AlN nucleation points continue to grow to form small islands through epitaxy, and then the small islands continue to merge and eventually form a smooth surface [25]. To date, the main method of III-nitride epitaxy on the surface of two-dimensional materials is to generate dangling bonds through plasma treatment, followed by epitaxially growing III-nitride materials. This method results in covalent bond formation between III-nitrides and two-dimensional materials, while it is not fully van der Waals epitaxy.

As previously reported, the improvement of the crystal quality of heteroepitaxial GaN film through the introduction of the AlN buffer layer was widely investigated [26,27,28]. However, there are few studies focused on how to improve the crystal quality of GaN on two-dimensional materials. In this work, we propose a pathway for the GaN epitaxy based on h-BN/sapphire substrates. A 25 nm thick AlN layer is deposited on the h-BN/sapphire substrate by magnetron sputtering, followed by the epitaxy process via a metal–organic chemical vapor deposition (MOCVD) system. After introducing the sputtered AlN layer, the surface morphology of GaN is greatly improved, and the crystal quality is promoted. Compared to the plasma treatment of h-BN, this method avoids the damage of h-BN.

## 2. Experiment

**Figure 1:**
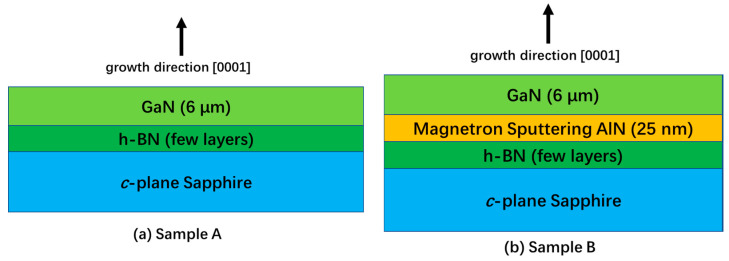
(**a**) GaN material epitaxially grown on h-BN/sapphire substrate of sample A and (**b**) GaN material epitaxially grow on h-BN/sapphire substrate of sample B after magnetron sputtering 25 nm thick AlN.

To obtain the h-BN/sapphire substrate, a few layers of h-BN are transferred from the copper foil to the *c*-plane sapphire substrate. The few layers of h-BN are commercial products (SixcarbonTech, Shenzhen, China), here, they are made on a Cu foil by low-pressure chemical vapor deposition. As shown in Figure 1, based on the h-BN/sapphire substrate, we grow 6 μm thick GaN films for sample A. Meanwhile, for sample B, the 25 nm thick AlN layer is sputtered through magnetron sputtering before GaN growth, and then 6 μm thick GaN is grown.

The AIXTRON CRIUS II MOCVD system [29] (Herzogenrath, Germany) is utilized for GaN epitaxial growth. The 25 nm thick nucleation layer is grown at 520 °C, in which the flow rate of NH_3_ is 12,500 mL/min, and the flow rate of TMGa is 75 mL/min. The pressure during the growth of the nucleation layer is 600 mbar. After that, the temperature is raised to 965 °C to start the growth of the 3D layer, the flow rate of NH_3_ is 24,000 mL/min, the flow rate of TMGa is 210 mL/min, the pressure is 300 mbar, and the total thickness is about 350 nm. Finally, the temperature is raised to 1080 °C for 2D layer growth, the flow rate of NH_3_ is 28,000 mL/min, the flow rate of TMGa is 440 mL/min, the pressure is 300 mbar, and the growth thickness is about 5.625 μm. ITOPS A320 equipment of North Huachuang (Beijing, China) is used for magnetron sputtering. For sample B, the Al target is bombarded with Ar at 600 °C, and Al ions are sputtered and combine with N ions to generate AlN, which is then deposited on the surface of the h-BN/sapphire substrate. In order to systematically investigate the samples, various measurements are performed. Scanning electron microscope (SEM) images are taken with a FEI MLA650F system (Hillsboro, OR, USA) at an accelerated voltage of 25 kV, and the magnification is 8288×. Transmission electron microscopy (TEM) investigations are performed with a Talos F200X (Thermo Fisher, Waltham, MA, USA), operated at 200 kV. The detailed surface morphology of the samples is further characterized by an atomic force microscope (AFM) under tapping mode in a Bruker ICON Dimension system (Billerica, MA, USA). Optical properties are obtained by a photoluminescence (PL) spectrometer, 2-679HR-HR 800 (Horiba, Palazzo, France), with a He-Cd laser operating at 325 nm. Additionally, the Raman spectra are achieved by Jobin Yvon LanRam HR800 (Horiba, Palazzo, France) with the excitation wavelength at 514 nm. X-ray diffraction measurements are performed in an X’Pert PRO (PANalytical, Almelo, Holland) system using the Cu-K line system with a wavelength at 0.154 nm. Finally, we obtain the X-ray photoelectron spectrometer (XPS) results with a ESCALAB 250Xi (Thermo Fisher, Waltham, MA, USA) system using Al-Kα radiation (λ = 1486.6 eV).

## 3. Results and Discussion

**Figure 2:**
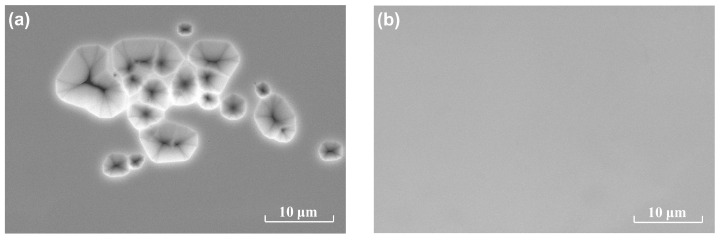
SEM images of (**a**) sample A and (**b**) sample B.

Both samples A and B are investigated by SEM to observe the surface topography of the GaN film. As shown in Figure 2a, there are many pits of different sizes in sample A. Since there are no dangling bonds on the surface of h-BN, GaN is difficult to nucleate on its surface. The low nucleation density results in sparse GaN islands and the formation of a smooth and continuous GaN film is difficult or even not possible [22]. As shown in Figure 2b, after 25 nm thick AlN on the surface of h-BN is sputtered, the problem of GaN nucleation on the surface of h-BN is alleviated. A smooth and continuous GaN film is obtained for sample B.

**Figure 3:**
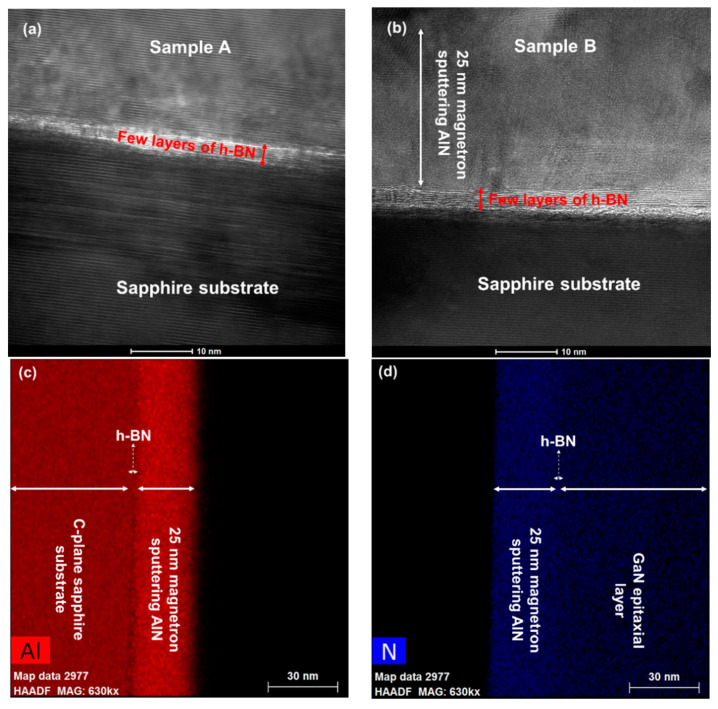
High-resolution TEM cross-sectional view of (**a**) sample A and (**b**) sample B. Energy dispersive spectroscopy (EDS) diagrams of (**c**) Al element and (**d**) N element.

To further investigate the two samples, Figure 3a,b show high-resolution TEM cross-sectional images of samples A and B, respectively. We can clearly observe the few layers of h-BN with a thickness of approximately 4 nm located on the sapphire substrate. In Figure 3a, the upper layer of h-BN is GaN. In Figure 3b, above the few layers of h-BN is 25 nm thick AlN, and GaN is above the AlN layer. Figure 3c,d are the EDS diagrams of Al and N. There is AlN about 25 nm thick on the h-BN/sapphire substrate, which proves the existence of 25 nm sputtered AlN in sample B.

**Figure 4:**
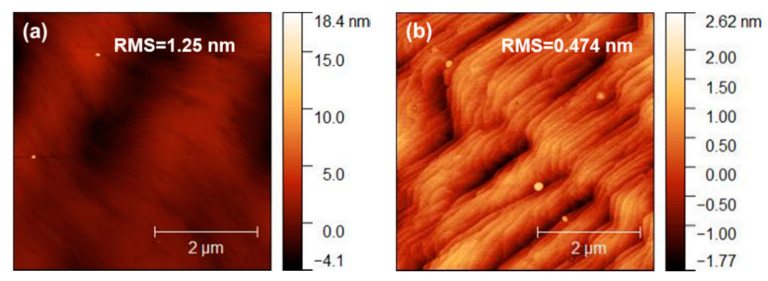
The 5 μm × 5 μm AFM images of (**a**) sample A and (**b**) sample B.

Figure 4 shows the AFM images of the two samples within 5 μm × 5 μm. Figure 4a is the AFM image of sample A, and the root mean square (RMS) roughness is 1.25 nm, while the RMS roughness of sample B is 0.474 nm. Obviously, sample B has less RMS roughness and a smoother surface, which is consistent with the SEM results shown in Figure 2.

**Figure 5:**
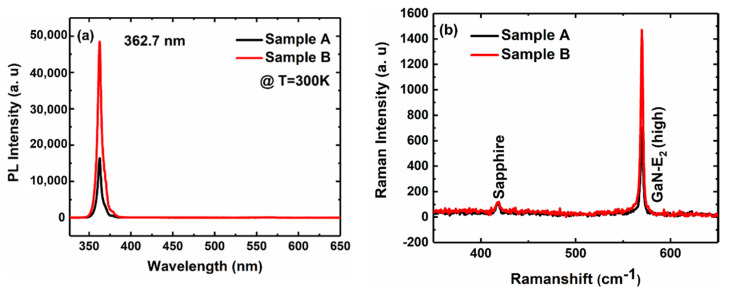
(**a**) PL and (**b**) Raman spectra of samples A and B at room temperature (300 K).

As shown in Figure 5, we further investigated the PL and Raman properties of the two samples. Figure 5a shows that the near band energy emission peaks of the two samples are both at 362.7 nm. The PL intensity of sample B is three times that of sample A, which indicates the improved crystal quality of sample B [30]. Figure 5b presents the Raman spectra of the two samples, in which the peak of the sapphire is located at 418.8 cm^−1^ and the E_2_ (high) peak of the two samples is located at 569.7 cm^−1^. The vibration mode of E_2_ (high) is most sensitive to stress, and the peak of E_2_ (high) for unstrained GaN is located at 567.6 cm^−1^ [31]. The E_2_ (high) peaks of both samples are located on the right side of 567.6 cm^−1^, indicating both samples are under a compressive stress state [32]. Besides, the intensity of the E_2_ (high) peak of sample B is 2.1 times higher than that of sample A, so the crystal quality of sample B is improved [33]. According to the results of PL and Raman measurements, the optical properties of sample B are better than sample A.

**Figure 6:**
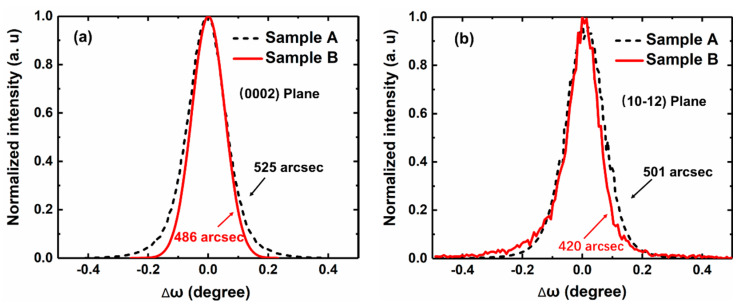
X-ray rocking curves of samples A and B on (**a**) (0002) and (**b**) (10–12) reflections.

To further evaluate the crystal quality of the two samples, we performed an X-ray rocking curve (XRC) measurement. As shown in Figure 6, the (0002)/(10–12) XRC full width at half maxima (FWHM) of samples A and B are 525/501 arcsec and 486/420 arcsec, respectively. The total dislocation density of sample A is 1.9 × 10^9^ cm^−2^, while sample B is 1.4 × 10^9^ cm^−2^, calculated from XRC results, which is 35.7% lower than that of sample A [34]. Therefore, sample B features better crystal quality [35], which is consistent with the optical measurements shown in Figure 5.

**Figure 7:**
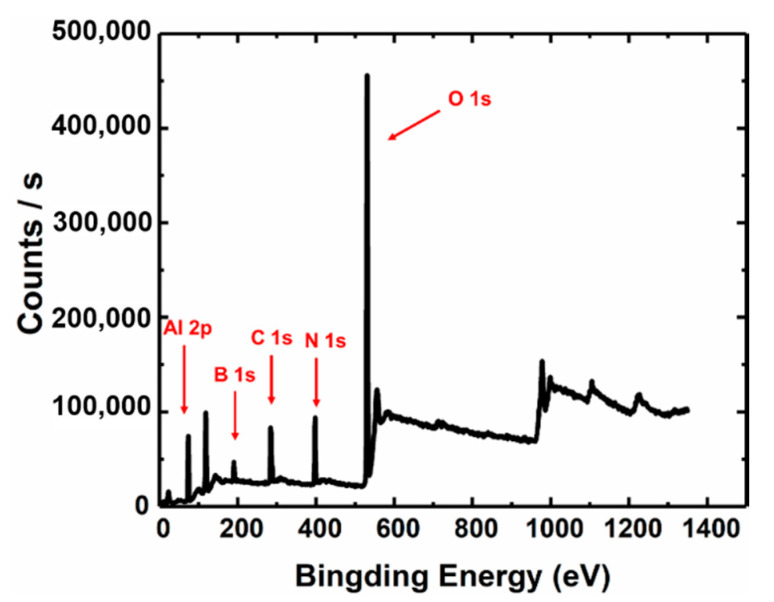
XPS results of h-BN/sapphire substrate.

Figure 7 is the XPS results of the h-BN/sapphire substrate. The B 1s peak and N 1s peak can be seen from the figure, and the presence of h-BN can be confirmed. The Al 2p peak and O 1s peak confirm the presence of the sapphire substrate. The C 1s peak comes from poly (methyl methacrylate) produced during the transfer of h-BN from copper foil to sapphire.

**Figure 8:**
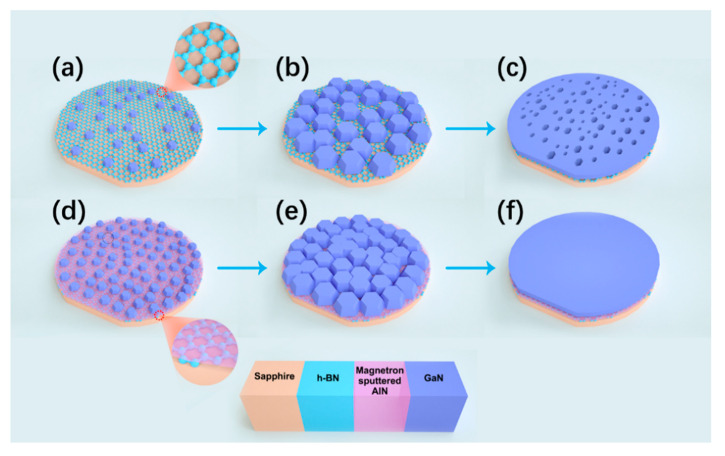
The epitaxial mechanism of GaN on h-BN/sapphire substrate and sputtered AlN/h-BN/sapphire substrate: (**a**) GaN nucleation process, (**b**) formation of GaN islands and (**c**) GaN film on h-BN/sapphire substrate; (**d**) GaN nucleation process, (**e**) formation of GaN islands and (**f**) GaN film on sputtered AlN/h-BN/sapphire substrate.

Based on the model of Wu et al. [23], we further explained the growth mechanism of samples A and B. Figure 8 shows the epitaxial mechanism of GaN on the h-BN/sapphire substrate and sputtered AlN/h-BN/sapphire substrate. Figure 8a shows the nucleation process of low-temperature GaN on the h-BN/sapphire substrate. Since there are no dangling bonds on the h-BN surface, the nucleation points are sparse and random. Figure 8d shows the nucleation process of low-temperature GaN on the sputtered AlN/h-BN/sapphire substrate. Due to the presence of the sputtered AlN buffer layer, GaN nucleation sites are dense. As the temperature increases, GaN nucleation islands gradually grow. Figure 8b,e show the GaN islands on the two substrates. Since GaN has denser nucleation points on the sputtered AlN/h-BN/sapphire, the number of GaN islands is also greater. Finally, the GaN islands merged and the two-dimensional growth begins. Figure 8c,f show the final GaN film. GaN does not form a smooth continuous film on the surface of the h-BN/sapphire substrate. There are many pits of different sizes on the GaN surface. In contrast, GaN epitaxially grown on sputtered AlN/h-BN/sapphire substrate has a continuous smooth film.

## 4. Conclusions

In summary, due to the lack of dangling bonds on the surface of h-BN, the direct growth of GaN on its surface will make it difficult for GaN to form a smooth and continuous film. We have proposed a pathway for epitaxial GaN on h-BN/sapphire substrates. A 25 nm thick AlN layer is deposited on top of the h-BN by magnetron sputtering, followed by the epitaxial growth of GaN via MOCVD. As a result, it is verified by SEM that the GaN surface is smooth, and the RMS roughness of the 5 μm × 5 μm AFM image is only 0.474 nm. According to the test results of PL and Raman spectra, the GaN epitaxy based on sputtered AlN/h-BN/sapphire substrate has better crystal quality, which is consistent with the results of XRCs. Finally, we explained the mechanism of sputtered AlN/h-BN/sapphire substrate epitaxial GaN through the schematic diagram, and further explained the effectiveness of our method. Our work provides a pathway for GaN epitaxy on the h-BN surface, which improves its surface morphology and crystal quality. Furthermore, high-quality III-nitride electronic devices and optoelectronic devices based on two-dimensional materials can be obtained through this method.
